# Inexpensive Apparatus for High-Quality Imaging of Microbial Growth on Agar Plates

**DOI:** 10.3389/fmicb.2021.689476

**Published:** 2021-06-30

**Authors:** Parker Smith, Martin Schuster

**Affiliations:** Department of Microbiology, Oregon State University, Corvallis, OR, United States

**Keywords:** imaging, colonies, agar plates, light box, apparatus, microbiology, inexpensive

## Abstract

The ability to capture images of results or processes is an important tool in the biologist’s tool kit. In microbiology, capturing high-quality images of microbial growth on agar plates is difficult due to the reflective surface of the plates and limitations in common photography techniques. Equipment is available to overcome these challenges, but acquisition costs are high. We have developed and tested an inexpensive and efficient apparatus for high-quality imaging of microbial colonies. The imaging box, as we have named the apparatus, is designed to eliminate glare and reduce reflections on the surface of the plate while providing uniform, diffuse light from all sides. The imaging box was used to capture hundreds of images in research and teaching lab settings.

## Introduction

Since its invention in 1839, photography has been utilized in science to document natural phenomena for later analysis (Wilder, 2009). In microbiology, high-quality images of microbial colonies on agar plates are important for research and education. Images can be used to preserve experimental results for further analysis and can be used as teaching material in lectures, textbooks, and online homework. It is difficult to capture quality images of colonies due to the nature of the agar plates. Petri dishes, whether glass or plastic, and the agar are reflective, but light is needed to capture images of colonies. This can result in a glare or a reflection appearing in the plate image, affecting the visibility of the colonies.

High quality images of plates can be captured using commercial apparatuses, but these are expensive (Table 1). They include stereo microscopes, gel imaging stations, and dedicated plate imagers such as the Singer PhenoBooth. Techniques developed by academic research groups make use either of high-resolution photo scanners converted for lab use (lab scanners, Table 1) or customized lighting apparatuses attached to a digital camera. Lab scanners are widely used for high throughput colony counting and growth analysis (Hartman and Tippery, 2004; Michel et al., 2008; Clarke et al., 2010; Levin-Reisman et al., 2014), although the mode of image capture through the bottom of the agar plate limits the resolution of morphological features. Other apparatuses have been custom-made for automated colony counting (Clarke et al., 2010; Brugger et al., 2012), imaging colonies in soft agar (Parkinson, 2007), or for imaging plates over time (Parkinson, 2007; Cobo et al., 2018; Reding et al., 2019). The specialization of these apparatuses increases the cost of construction as they include illuminators that provide transmitted light through the bottom of the plate (Parkinson, 2007; Clarke et al., 2010; Brugger et al., 2012), climate control equipment (Cobo et al., 2018; Reding et al., 2019), or fluorescence imaging capability (Reding et al., 2019).

**TABLE 1:** Comparison of the imaging box to other devices.

	Type	Cost^1^	Resolution	Color	Fluorescence
Imaging box (this study)	Plate imager	$33	Varies^2^	Yes	No
Singer PhenoBooth+	Plate imager	$22,000	23 MP	Yes	Yes
UVP Colony Doc-It	Plate imager	$7,660	17.9 MP	Yes	Yes
UVP BioDoc-It	Gel imaging system	$250–$13,000	1.3 MP	No	No
Canon Pixma Mg2522	Scanner	$99	600 dpi	Yes	No
Epson Perfection V39	Scanner	$99	4,800 dpi	Yes	No

*^1^The cost listed for the imaging box is the cost to construct the apparatus while the cost listed for commercial units is the cost to purchase new. The UVP BioDoc-It is no longer available new but is available used in a wide price range. ^2^The resolution of images captured using the Imaging Box depends on the camera being used. The cameras used in this study had resolutions between 10 and 12 megapixels.*

We have developed an inexpensive apparatus for capturing high-quality images of colonies on agar plates. The apparatus can be easily assembled from generic components in approximately 5 h with one overnight waiting step. The total costs for materials are approximately $33. Our system makes use of a light box that provides uniform, adjustable light through a diffuser, eliminates glare and reflections, and provides a uniform background. We have used this system to capture bacterial colony phenotypes in a transposon library screening project, and to capture images for teaching labs that have moved online as part of our university’s COVID-19 response in Spring 2020.

## Materials and Equipment

**FIGURE 1:**
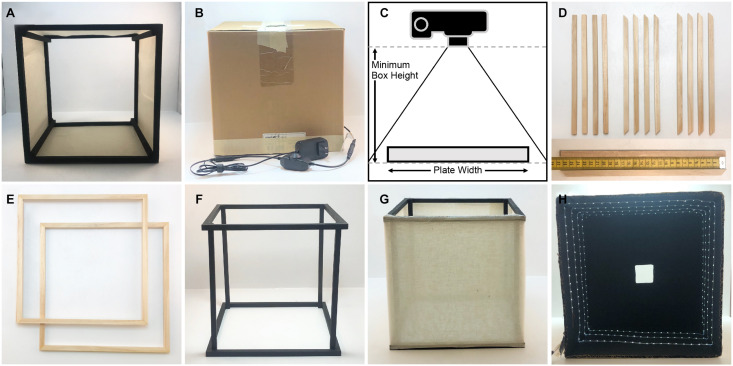
Components and construction of the imaging box. **(A)** Light diffusing component of the imaging box that is open on the top and the bottom (side view). **(B)** Light box component of the imaging box that eliminates unwanted light and glare while providing lighting for imaging. **(C)** Determining the necessary height for the imaging box. **(D)** Twelve square wooden dowels cut for the construction of the light diffuser, eight cut with 45° angles and four cut with 90° angles. The measuring tape on the bottom indicates dowel length in centimeters. **(E)** The top and bottom frames of the light diffuser constructed using the eight wooden dowels cut with 45° angles. **(F)** The assembled diffuser frame painted matte black. **(G)** The completely assembled diffuser. **(H)** The inside of the light box showing the LED light strip on the sides of the box, the matte black interior, and the square hole in the top of the box.

The imaging box is composed of two main parts, a light diffuser that is open on top and bottom (Figure 1A), and a light box that has internal lighting on all four sides, an open bottom, and a small camera hole for image capture on the top (Figure 1B). The diffuser is made with a cube frame. The frame is constructed out of 1 cm square, wooden dowels that are painted matte black with a thin, white fabric attached to the sides of the frame to diffuse the light. The fabric must be thin enough to transmit some of the light without producing a discernable light pattern. The craft fabric we chose was selected by shining a flashlight through several candidate fabrics and choosing the one that provided the best results. The light box is made from a square, sturdy cardboard box that is slightly larger than the diffuser frame, open on the bottom, and has a hole cut in the center of the top side. The inside of the box is painted matte black to prevent glare and reflections on the plates that are being imaged, and a strip of bright white (6,000 K) light-emitting diodes (LED lights) is attached to the inside of the box on the sides in a spiral pattern. The parts required to construct the box and their costs are listed in Table 2.

**TABLE 2:** Materials for the construction of the imaging box.

Item	Dimensions/amount used^1^	Cost	Manufacturer (Source)
LED strip light	1 unit, 4.45 m long (207 surface mounted diode [smd] 2835 LEDs)	$16.99	Lighting Ever, Las Vegas, Nevada (Amazon)
Square dowel	4 pieces, 9.5 mm diameter, 91.4 cm long	$1.10 each	Woodgrain, Fruitland, Idaho (Home Depot)
Fabric	110 cm × 26 cm	$3.27 per meter	Fairfield, Danbury, Connecticut (Hobby Lobby)
Black primer	340 g	$4	Rust-Oleum Corporation, Vernon Hills, Illinois (Walmart)
Wood glue	237 ml	$4–$7	Titebond, Columbus, Ohio (Home Depot)
Black poster board	56 cm × 71 cm	$0.69	Pen + Gear, Bentonville, Arkansas (Walmart)

*^1^Standard metric units provided throughout.*

## Methods

### Construction

The dimensions of the box are determined by the camera and lens type available, and by the size and shape of the agar plate that will be imaged. We built our box for imaging individual 10 and 15 cm diameter agar plates with an off-the-shelf point-and-shoot digital camera (Canon PowerShot A1000IS). To determine the width of our diffuser box, we added roughly 10 cm to the width of our plate. To empirically determine the necessary height, we measured the minimum height at which the camera can capture the entire plate without using the zoom function (Figure 1C). Our light diffuser is a 26 cm cube. The top and bottom of the diffuser frame are constructed from eight square wooden dowel pieces that are cut with 45° angles on each end and are 26 cm on the long side, and four wooden dowel pieces cut with 90° angles that are 26 cm long for the vertical supports (Figure 1D). Four of the trapezoidal dowels are glued together at the 45° joints using wood glue to construct a square frame for the top (Figure 1E). The other four trapezoidal dowels are glued together in the same way to construct the frame for the bottom. The vertical supports are glued inside the square frames (Figure 1F). This frame is painted matte black and then fabric is attached to the sides using staples (Figure 1G). We have experimented with both a single layer of a thin, white, interfacing fabric, and a double layer of white cheesecloth. Both provide efficient light scattering to produce diffused light within the box.

The light box needs to be slightly larger than the diffuser frame so it fits comfortably over it. We chose to use a cardboard box that was 31 cm square measured on the outside, and 0.5 cm thick. We cut the box so that it was open on the bottom and was 29 cm tall. A 5 cm square hole is cut in the center of the top of the box. The inside of the box is painted matte black. A 445 cm strip of LED lights is attached to the inside walls of the box in a spiral fashion so that each side has at least 3 horizontal lines of LED lights on it (Figure 1H). We chose a specific, cuttable strip light with an adhesive backing for easy installation (see Table 2). An attached dimmer switch allows control of the brightness inside the box. A small hole is punched in the side of the box close to the bottom where the plug is threaded through. The LED lights are attached starting from this point working upward toward the top of the box. The backdrop underneath the plates on the bottom can be matte black or matte white; we have found that a piece of poster board provides good results.

### Imaging for Research and Teaching Projects

Freshly grown microbial specimen on agar plates were placed inside the imaging box. Images were captured either with a hand-held Canon Powershot A1000IS, using an automated acquisition setting (P, Program mode) and auto-focusing, or with cell phone cameras. Image files were visualized in Adobe Illustrator without any image manipulation.

### Validation

To validate the performance of the imaging apparatus, we used a higher-end digital single-lens reflex (DSLR) camera (Canon EOS Rebel XSi) attached to a camera rig. The rig standardized distance of the camera from the plate, improved stability, and allowed the image taker to move away from the camera so that their reflection did not show up on the plate images captured without the imaging box. To determine the suitability of the imaging box for taking glare-free images, we compared the glare of images taken with and without the apparatus. The images of an empty MacConkey agar plate and a lysogeny broth (LB) agar replica plate were captured with the camera set to Program mode. For each plate three images were captured, one with the imaging box, one without the imaging box at the lab bench, which is directly under a light, and one without the imaging box, but with the location optimized to reduce glare as much as possible. The latter condition represents the typical setting for capturing images without some sort of apparatus. The glare was quantified with the imaging software ImageJ (Schneider et al., 2012) by drawing a straight line across the plate and generating a profile plot, and also by drawing a rectangle on the surface of the plate and generating a three-dimensional surface plot. For the MacConkey agar plate the line was drawn across the middle of the plate, and for the replica plate the line was drawn through the middle of a row of colonies. For both plates the rectangle was drawn in the center of the plate so as to not hit the edges of the plate. For the profile plot, red, green and blue (RGB) pixel intensities are reported. For the three-dimensional surface plots, the RGB pixel intensities were converted to grayscale in ImageJ using the formula *V* = *(R* + *G* + *B)/3* to report a single “brightness” value. To quantify the reduction in glare, the standard deviation of the brightness for the pixels in the area used for the three-dimensional surface plot was calculated.

To compare the quality of images captured with the imaging box to commercially available apparatuses, we captured images with the imaging box, a Canon Pixma Mg2522 printer/scanner, and a UVP BioDoc-It gel imaging station. Images were captured of an empty MacConkey agar plate, an LB plate with *E. coli* DH5-α pSW002-*P*_psbA_-DsRed-Express2 (Wilton et al., 2018), and an LB plate with *E*. *coli* DH5-α pSW002-*P*_psbA_-E2-Crimson (Wilton et al., 2018). A different batch of MacConkey agar plates was used for this experiment. Images taken with the imaging box were captured as described above with the DSLR camera in Program mode. Images taken with the Canon Pixma Mg2522 were captured using a photo scan set to 600 dpi. The plates were placed face up, with the agar side on the surface of the scanner with a matte black background placed above it as described previously (Levin-Reisman et al., 2014). Images taken with the BioDoc-It imaging station were captured with the white light set to high, the UV light turned off, and the image acquisition time set to 1 s. To compare the quality of the images, the standard deviation of the brightness of a square in the middle of the MacConkey plate was calculated in ImageJ. To ensure that the same area of the plate was being analyzed for the different imaging apparatuses, a 5 cm square was drawn on the bottom of the plate with a white paint pen. This was done to control for the fact that the different imaging apparatuses generate different sized images with different pixel counts, making it impossible to use the pixel readings in ImageJ to compare the same area between photos. The area inside of the square was analyzed taking care to exclude all of the white lines.

To evaluate the consistency of images taken with the imaging box over a period of time, an imaging standard was photographed every 24 h for 1 week, using identical camera settings. The focus here was on the technical performance of the box over time, specifically the consistency in lighting conditions, when images are taken by the same user with the same camera. The focus was not on evaluating the consistency of images taken by different users or with different camera types. These images were captured in quadruplicate with the camera in the manual exposure mode with the shutter speed set to 1/50th of a second, the aperture set to 5.6, and the ISO speed set to 200. Two identical imaging standards were used with each set of four images consisting of two images of each standard. The imaging standards were stored in the dark when they were not in use to prevent photobleaching. We quantified image quality with ImageJ (Schneider et al., 2012) by drawing a box in the white region of the imaging standard and measuring the brightness. The RGB intensities of the pixels were converted to brightness values using the formula above. The mean brightness of each square was calculated and graphed as a percentage value. During this experiment, the dimmer was removed from the Imaging Box so that the light levels would remain consistently high throughout the experiment. To assess the sensitivity of this approach, images were taken using the same methods but with the dimmer set to 1 (fully on), 3/4, 1/2, and 1/4. Fractions indicate the relative distance between the fully on and the fully off position. For statistical analysis, a One-way ANOVA was performed using GraphPad Prism (version 6.00 for Windows, GraphPad Software, La Jolla, CA, United States). In case of a statistically significant difference (α = 0.05), a Tukey’s multiple comparison test was performed.

## Results

### Validation of Image Quality

**FIGURE 2:**
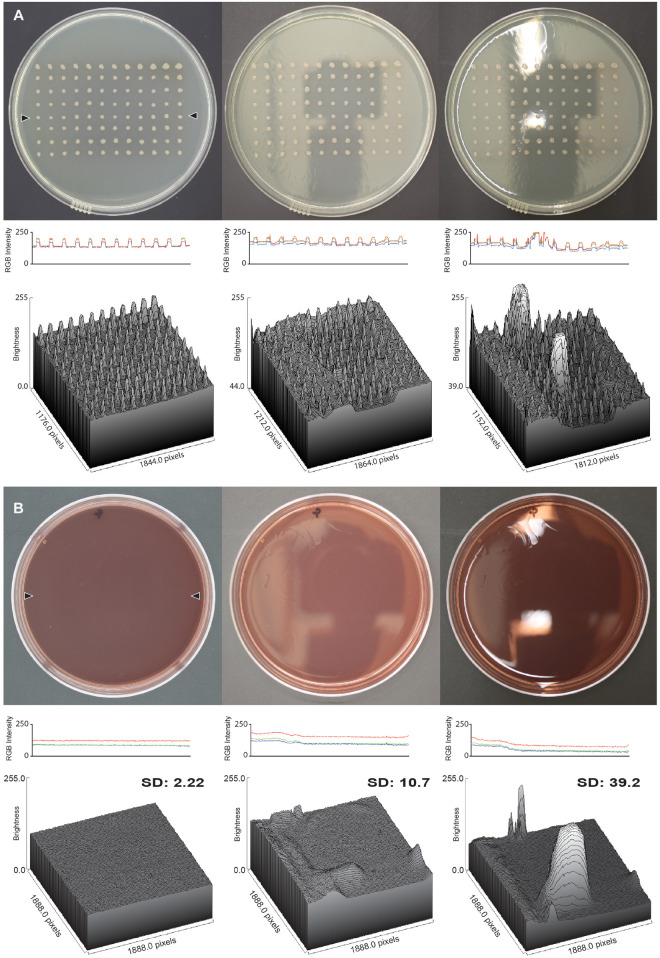
Glare reduction in images captured with the imaging box. **(A)** Analysis of an LB replica plate with colonies of *P. aeruginosa*, and **(B)** an empty MacConkey agar plate, in both cases imaged in the imaging box (left column), without the imaging box but in an optimized location (center column), and without the imaging box on the lab bench (right column). The middle row shows the RGB profile plot of the images in the top row. The plot measures the RGB intensity of a line of pixels drawn across the plates between the locations marked by the two black triangles in the image captured with the imaging box. The line for the RGB profile plot was drawn in the same spot on all three plates, and the analysis was carried out in ImageJ. The bottom row shows the three-dimensional surface plot of pixel brightness of a grayscale image from a rectangular area that captured as much surface area on the plate as possible without including the edge of the plate. The standard deviation of the brightness is shown for each surface plot.

Once the apparatus was constructed, we validated its suitability for taking glare-free images of consistently high quality. First, we compared the glare of images of two different agar plate types taken with and without the apparatus (Figure 2). The imaging box produced images that have little to no glare when compared to images taken without the imaging box. The image captured of an LB replica plate with the light box shows a uniform background level of light intensity from which colonies stand out as crisp, defined peaks in both a profile plot and a three-dimensional surface plot (Figure 2A, left panels). The image captured of the replica plate without the imaging box from the lab bench directly under the light shows the most glare with spikes of brightness on both the profile and the surface plot, potentially obscuring features of the colonies in the image (Figure 2A, right panels). The image captured of the replica plate without the imaging box but in an optimized location shows little glare, but shows variations in the levels of background light intensity across the plate as a result of the reflections of the ceiling on the surface of the plate (Figure 2A, center panels). The results for the images captured of an empty MacConkey agar plate are similar to those seen for the replica plate (Figure 2B). The image captured in the imaging box has the least amount of glare while the image captured without the imaging box on the lab bench has the most glare. The lack of colonies on this plate makes it easy to see the consistency of the background light intensity levels in both profile and surface plots when comparing the image captured in the imaging box to the images captured without it (Figure 2B). The observed differences are reflected in the standard deviations of the brightness calculated for each MacConkey agar surface plot.

Second, we compared our imaging box to two other devices commonly used to image colonies on plates, a scanner and a gel imaging station. We photographed an empty MacConkey agar plate, and two plates of *E. coli* expressing the red fluorescent protein variants DsRed-Express 2 and E2-Crimson, respectively (Supplementary Figure 1). The standard deviations of the brightness values derived from rectangular surface plots were very similar in all three cases. However, there were some noticeable differences as well. The scanner produced colonies that are less-well defined and less intense, because they are imaged through the agar. The gel imaging station captured plate images that show some glare around the edges, albeit outside the quantified area. The particular gel imaging station available to us also only produces black and white images, whereas newer models do provide a color mode.

**FIGURE 3:**
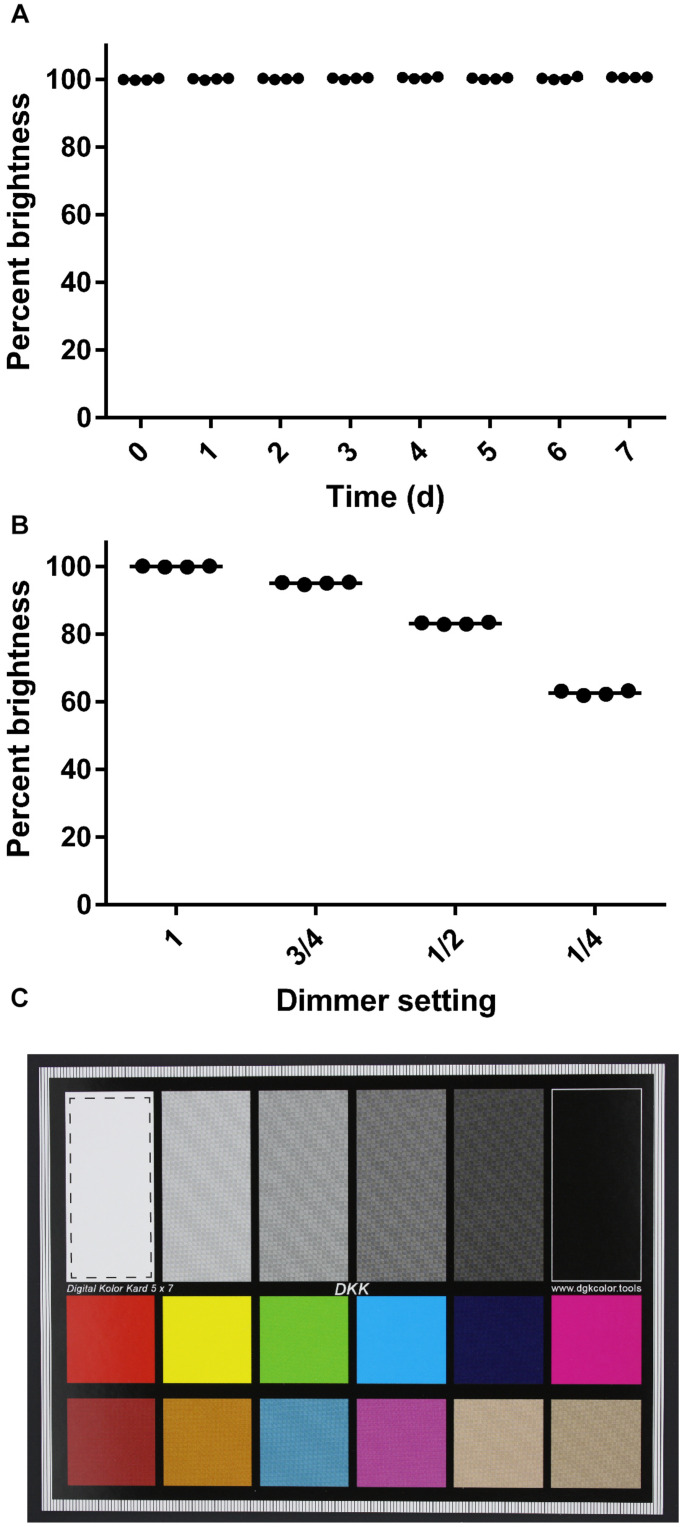
Consistency of images captured with the imaging box. **(A)** Percent brightness of an image standard over a 7-day time course. The dimmer was removed from the apparatus for this series. Dots represent individual replicates (*n* = 4), and the bars represent means. The first time point is set to 100% brightness. **(B)** Percent brightness of an image standard captured at different dimmer settings. Dots represent individual replicates (*n* = 4), and the bars represent means. The brightness value obtained at the maximum dimmer setting is scaled to 100%. **(C)** Image standard used. The dashed line box in the white rectangle shows the area that was analyzed for mean brightness values in ImageJ.

Third, we evaluated the consistency of images taken with the imaging box over a period of time (Figure 3). We photographed an imaging standard (Figure 3C) every 24 h for 1 week, using identical camera settings. Over the course of this experiment, we saw no significant change in the brightness of the images captured of an imaging standard (One-way ANOVA, *p* > 0.05; Figure 3A). We verified the sensitivity of this experiment by capturing images of the same imaging standard at different dimmer settings (Figure 3B). We found a significant difference in the percent maximal brightness between the images taken at the highest setting and incrementally lower settings (One-way ANOVA, *p* < 0.001).

### Applications in Research and Teaching

**FIGURE 4:**
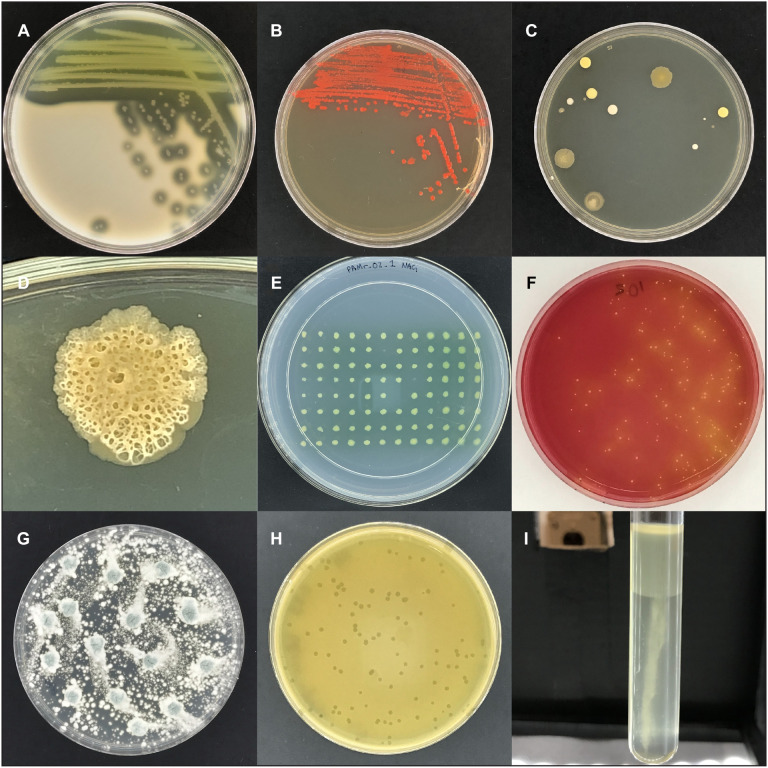
Images captured with the imaging box. **(A)** Area of clearing on skim milk plates caused by protease secretion of *P. aeruginosa*. **(B)** Colonies of *E. coli* labeled with the red fluorescent protein DsRed-Express2. **(C)** Colonies of unknown microbes on an agar plate that was left open to the environment. **(D)** Close-up image of an unknown microbial colony. **(E)** Replica plate from the *P. aeruginosa* transposon mutant screening experiment. The three positions without a colony (B6, D8, and E7) represent non-growing mutant candidates. **(F)** A potato salad sample plated on mannitol salt plates to detect mannitol fermenting *S. aureus*, indicative as yellow colonies with yellow zones. **(G)** A plate of *Penicillium sp.* **(H)** Bacteriophage c2 plaques in a lawn of *Lactococcus lactis*. **(I)** Growth of *Proteus vulgaris*, a facultative anaerobe, in oxygen-reducing sodium thioglycolate medium, with dense growth in the high oxygen region and sparse growth in the low oxygen region.

The design for the imaging box was borne out of a desire to capture pictures of the bacteria we work with in the laboratory, for scientific research, for undergraduate teaching, and for public outreach. In the lab we see the amazing diversity of bacterial colony morphologies on agar plates on a daily basis, whether it is the proteolysis of skim milk by *Pseudomonas aeruginosa* (Figure 4A), fluorescently labeled *Escherichia coli* (Figure 4B), or unknown contaminants on a plate that was left uncovered (Figures 4C,D).

Specifically, we used the imaging box for two different projects after its completion. In the research lab we used the imaging box to document the results of a phenotype screening project. The project employed a non-redundant transposon mutant library of the opportunistic pathogen *P. aeruginosa*, available as 5,600 individual strains arrayed in sixty-three 96-well plates (Liberati et al., 2006). Previously we had identified N-acetyl-L-glutamic acid (NAG) as a carbon or nitrogen source that a wild type strain can utilize but a mutant deficient in the stationary-phase sigma factor RpoS cannot (Robinson et al., 2020). We screened the mutant library to identify the RpoS-regulated gene(s) responsible for the observed phenotype. Using a 96-well replica plater, we plated the transposon library on solid minimal medium with NAG as the sole nitrogen source (Figure 4E), archiving images of all 5,600 *P. aeruginosa* mutant strains. The experiment was conducted using sets of 10 plates at a time, with the imaging box making it possible to capture individual images of all 10 plates in less than three and a half minutes. Over the course of the experiment we identified several candidate mutants (Figure 4E). Follow-up experiments will include the elimination of false-negatives, the discrimination between NAG deficiency and general amino acid auxotrophy, and ultimately their genetic and biochemical characterization.

The second project involved the generation of teaching materials for a remote microbiology lab classes at Oregon State University. In response to the COVID-19 pandemic all classes at Oregon State University were delivered remotely during the 2020 spring term. For the microbiology lab classes this meant that labs had to quickly be switched from an in-person to a remote lab experience in which agar plate results were shared as digital images. The light box was used by instructors to build a large collection of high-quality images. These included experiments in food contamination, fungal growth, phage biology, and bacterial physiology. A sample of potato salad was plated on mannitol salt agar to test for contamination by *Staphylococcus aureus* (Figure 4F). A *Penicillium* sp. culture was grown as an example of fungal growth (Figure 4G). A sample of bacteriophage c2 was plated in a lawn of *Lactococcus lactis* to determine the phage titer (Figure 4H). Several strains of bacteria were inoculated in oxygen-reducing sodium thioglycolate medium to determine the oxygen requirements of the strains (Figure 4I). The apparatus withstood such repeated, high-volume usage with no decrease in performance.

## Discussion

In conclusion, we designed our imaging box using components that are cost efficient, available, and durable enough to hold up to heavy usage. We verified the high quality and consistency of the images taken, and we demonstrated the utility of the apparatus in research and teaching lab projects.

Most of the materials that were used in the construction of the imaging box can be substituted with alternative materials that may be more readily available or may be better-suited for the specific application. For example, we experimented with using both a white craft fabric and a double layer of cheese cloth as a diffuser, but any thin white fabric that does not produce a light/shadow pattern in the transmitted light could be used. We constructed our diffuser frame out of wood, but it could also be constructed out of metal, plastic, or even another cardboard box with large windows cut in the sides. We choose to make the light box component out of cardboard, because we happened to have a heavy-duty box available that was of the exact size needed for construction. A box constructed out of plywood would also work well but would require additional funds and construction time.

While using the imaging box in the above-mentioned experiments, we have found that the simplicity of the design gives rise to some challenges that need to be considered, but can be worked around. The primary challenge is the size of the imaging aperture cut into the top of the light box. We cut our hole for use with a specific camera and later found that taking images with different cameras or cell phones resulted in light pollution in the box that caused a glare to appear on plate images. This can be addressed by using a piece of black paper to cover any gaps between the camera and the box that allow light to get in. In the future our plan is to 3D-print adapters for different-sized cameras that will ensure a tight fit with no light pollution. Another issue with the current design is that it is intended for capturing plate images directly from above. If plate images from a side angle are needed, modifications to the apparatus would need to be made. We have found that side angle images of test tubes can be taken by placing the apparatus on its side and imaging through the open bottom.

Taken together, the appeal of our apparatus’ design is in its simplicity and cost efficiency. It enables capturing quality images of bacterial colonies on solid agar plates for roughly $33 using readily available materials and modest construction time. As such, it is likely to find broad application in the microbial sciences.

## Data Availability Statement

The raw data supporting the conclusions of this article will be made available by the authors, without undue reservation.

## Author Contributions

PS conceived of and designed the study, acquired and analyzed images, and wrote the first manuscript draft. MS contributed to study design, edited the manuscript, and acquired funding. Both authors contributed to manuscript revision, read, and approved the submitted version.

## Conflict of Interest

The authors declare that the research was conducted in the absence of any commercial or financial relationships that could be construed as a potential conflict of interest.
