# Supplementary material for: Inexpensive Apparatus for High-Quality Imaging of Microbial Growth on Agar Plates
Source: Front Microbiol. 2021 Jun 30;12:689476. doi: 10.3389/fmicb.2021.689476 (PMC8278329; doi:10.3389/fmicb.2021.689476)
Supplement: Supplementary file 1 [file Image_1.pdf]

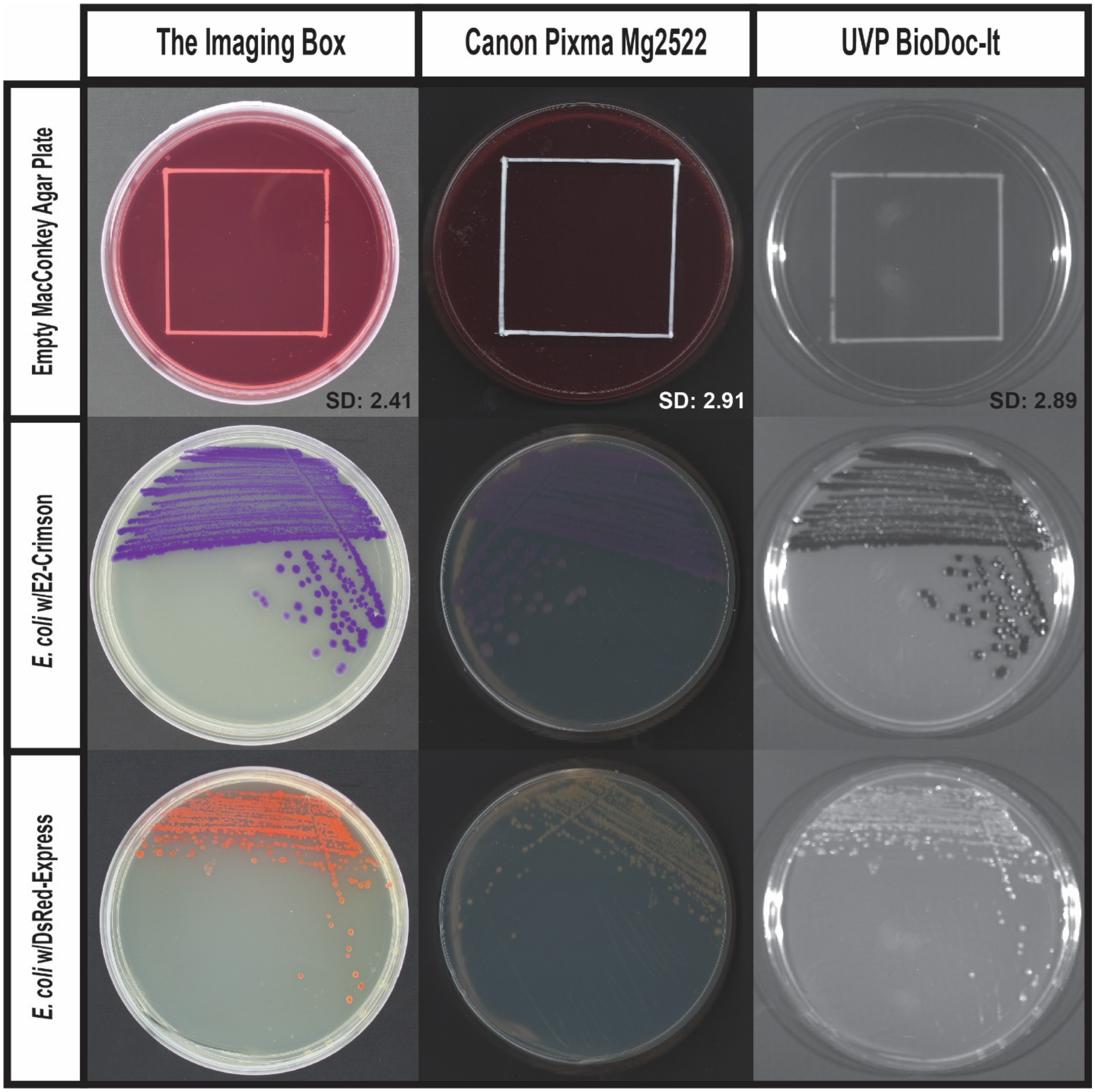

Supplementary Figure 1. Comparing the imaging box to other apparatuses. Images of plates were captured using the imaging box (left column), a Canon Pixma MG2522 scanner (middle column), and a UVP BioDoc-It gel imaging apparatus (right column). Images were captured of an empty MacConkey agar plate (top row), a culture of *E. coli* DH5-α pSW002-P_{psbA}-E2-Crimson on an LB agar plate (middle row), and a culture of *E. coli* DH5-α pSW002-P_{psbA}-DsRed-Express2 on an LB agar plate (bottom row). The standard deviation of the brightness of pixels inside the white square drawn on the MacConkey plate was calculated using ImageJ and is displayed in the bottom right hand corner of the MacConkey plate images.
